# Burst kinetics and CNNM binding are evolutionarily conserved properties of phosphatases of regenerating liver

**DOI:** 10.1016/j.jbc.2023.103055

**Published:** 2023-02-22

**Authors:** Rayan Fakih, Robert H. Goldstein, Guennadi Kozlov, Kalle Gehring

**Affiliations:** Department of Biochemistry, Centre for Structural Biology, McGill University, Montreal, Quebec, Canada

**Keywords:** cell signaling, protein phosphatase, dual-specificity phosphoprotein phosphatase, X-ray crystallography, analytical ultracentrifugation, isothermal titration calorimetry, enzyme kinetics, phosphocysteine, UEX protein

## Abstract

Phosphatases of regenerating liver (PRL or PTP4A) are a family of enigmatic protein phosphatases implicated in cell growth and metabolism. Despite their relevance in metastatic cancer, much remains unknown about the PRL family. They act as pseudophosphatases to regulate the CNNM family of magnesium transporters yet also have enzymatic activity on unknown substrates. In mammals, PRLs are mostly found trapped in an intermediate state that regulates their pseudophosphatase activity. Phosphocysteine, which is formed as an intermediate in the phosphatase catalytic cycle, is inefficiently hydrolyzed leading to burst enzyme kinetics and turnover numbers of less than one per hour. In flies, PRLs have recently been shown to have neuroprotective and neurodevelopmental roles raising the question whether they act as phosphatases, pseudophosphatases, or both. Here, we characterize the evolutionary development of PRLs and ask whether their unique structural and functional properties are conserved. We purified recombinant PRL proteins from 15 phylogenetically diverse organisms and characterized their catalytic activities and ability to bind CNNM proteins. We observed PRLs from humans to amoebae form a stable phosphocysteine intermediate and exhibit burst kinetics. Isothermal titration calorimetry experiments confirmed that the PRL–CNNM interaction is broadly conserved with nanomolar affinity in vertebrates. Lastly, we determined the crystal structure of the *Drosophila melanogaster* PRL–CNNM complex and identified mutants that specifically impair either phosphatase activity or CNNM binding. Our results reveal the unique properties of PRLs are conserved throughout the animal kingdom and open the door to using model organisms to dissect PRL function in cell signaling.

Phosphatases of regenerating liver (PRL1, also known as PTP4A) are a small family of membrane-bound cysteine phosphatases within the CC1 (cysteine-based, group 1) protein phosphatase superfamily. PRLs were first studied by Diamond *et al.* (1) who discovered that PRL1 was strongly upregulated in regenerating rat liver and contributed to regulating cell growth as a potential oncogene. PRL3 was later shown to be a critical factor for metastasis of colorectal cancer (2). PRLs are now considered to be the most oncogenic protein phosphatases and the target of broad efforts for the development of anticancer inhibitors (3, 4, 5, 6, 7).

PRLs are single domain phosphatases with conserved WPD and P-loops typical of CC1 phosphatases (Fig. S1). At their C-terminus, PRLs possess a prenylation motif (CAAX box), which directs their association with Golgi, early endosome, and plasma membranes in cells (reviewed in (3, 8)). PRL phosphatase activity is regulated by a variety of chemical modifications of the catalytic cysteine (9). The cysteine readily forms a disulfide with an adjacent cysteine even in the presence of low concentrations of reducing agents (10). The cysteine also becomes phosphorylated as part of the catalytic cycle. A phosphorous-sulfur intermediate occurs in all cysteine-based phosphatases (11, 12), but the intermediate is unusually long-lived in PRLs (10, 13, 14). The accumulation of phosphorylated enzyme leads to the phenomenon of burst kinetics, which is characterized by a fast initial rate of catalysis followed by a slow steady-state rate (10, 15). In cells, PRLs are largely found in the phosphorylated form and the level of phosphorylation varies as a function of magnesium availability (13, 14). The substrate responsible for the phosphorylation *in vivo* remains unknown. *In vitro*, the PRL cysteine becomes partially phosphorylated in the presence of ATP and other phosphate-containing compounds (16).

A key step in understanding the physiological function of PRLs came with the identification of their interaction with CNNM proteins (17, 18). CNNM Mg^2+^ transporters are important actors of ion homeostasis in cells and mutated in genetic diseases associated with Mg^2+^ uptake and retention (19). In eukaryotes, CNNM activity is regulated by the binding of PRLs (17, 18). Unbound CNNMs are capable of translocating Mg^2+^ out of the cell, while PRL–CNNM complexes are not (13, 14, 17, 20). The oncogenicity of PRLs is thought to depend on their binding and inhibition of CNNMs (14, 17, 18).

Multiple structures of PRLs alone and bound to CNNM proteins have been determined (10, 13, 14, 16, 21, 22, 23, 24). PRLs bind to the cystathionine-β-synthase (CBS)-pair (Bateman) domains of CNNMs. A CNNM aspartic acid residue inserts into the PRL catalytic site, mimicking a phosphosubstrate and inhibiting PRL activity (13). Although not in direct contact with the aspartic acid, the PRL catalytic cysteine plays a key role in the interaction and binding is strongly decreased by oxidation, phosphorylation, or mutation of the cysteine (13). Testing of a mutation that specifically blocks phosphatase activity showed that CNNM binding was sufficient for PRL oncogenicity in a mouse model of metastasis but left unanswered the question of the function of phosphatase activity in PRL signaling (14).

To gain insight into the evolutionary conservation of the structural and functional properties of PRLs, we studied the proteins from a range of eukaryotic phyla. We found that burst kinetics is broadly conserved but with a large range in the stability of the phosphocysteine intermediate. We also examined the ability of selected PRLs to bind CNNM proteins and determined the crystal structure of the PRL-CNNM CBS-pair complex from fruit flies. This allowed the characterization of the mutants of fly PRL that specifically block either CNNM binding or phosphatase activity.

## Results

### PRL phylogeny and species selection

We used the NCBI BLAST to identify PRL homologs from across the spectrum of eukaryotic lineages: Excavata (Heterolobesea, Euglenozoa), Chromalveolata (Heterokonta, Alveolata, Foraminifera), Amorphea (Amoebozoa, Fungi, Ichthyosporea, Filasterea, Choanoflagellatea, Animalia), and plants (Rhodophyta) (Fig. S1 and Table S1). PRL sequences were differentiated from the closely related CDC14 tyrosine phosphatases based on the presence of a C-terminal prenylation motif and an alanine following the catalytic arginine in the P-loop. Nearly all CC1 phosphatases have a serine or threonine at this position that participates in hydrolysis of the phosphocysteine intermediate (25, 26). PRLs are absent in the Fornicata (Trichozoa) and Parabasalia groups. Among plants, PRLs are conserved in the Rhodophyta group (red algae and related seaweeds) but are absent in Viridiplantae (green algae and land plants). We selected multiple PRLs from the phylum Euglenozoa because of their relevance for human diseases. The selected PRLs were cloned without the prenylation site and the recombinant proteins expressed and purified for structural and functional characterization. In total, 17 PRL proteins were prepared from 14 distinct model organisms including five from kinetoplastid parasites (Table S2).

### A long-lived phosphocysteine intermediate is a conserved feature of PRLs

**Figure 1:**
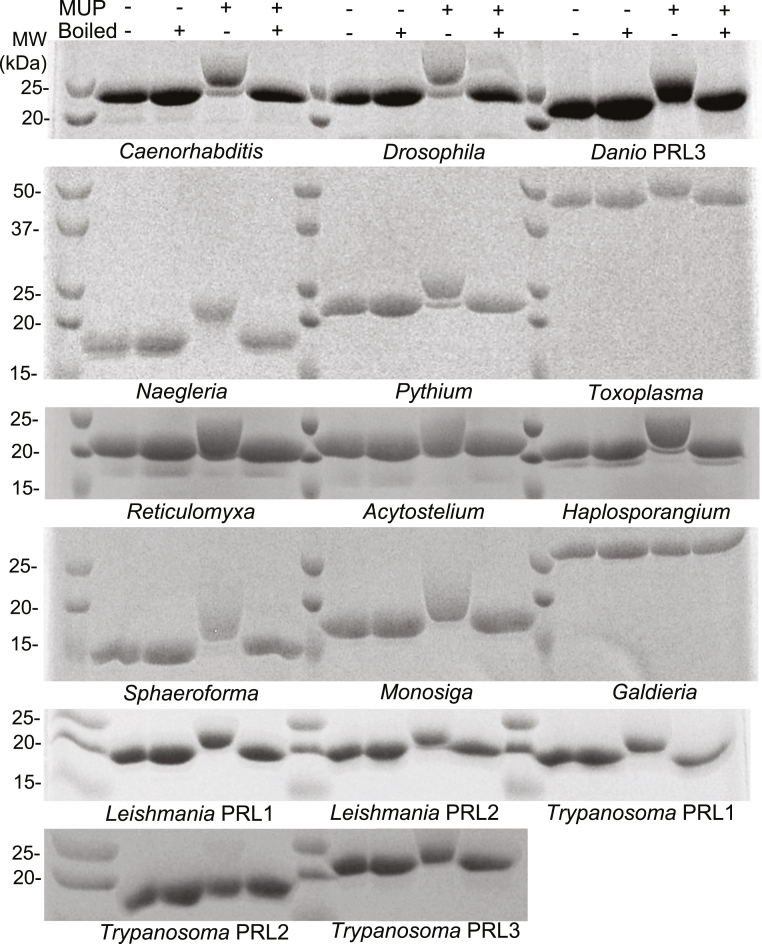
**Formation of a long-lived phosphocysteine intermediate is evolutionarily conserved.** Recombinant PRLs were incubated with or without a synthetic substrate, 4-methylumbelliferyl phosphate (MUP), and analyzed on Coomassie-stained, Phos-tag gels with or without boiling to hydrolyze phosphocysteine. All the samples, except *Galdieria* and *Trypanosoma* PRL2, showed the presence of a slower migrating, phosphorylated protein band in the presence of MUP. Two of the PRLs, *Toxoplasma* and *Galdieria*, have higher molecular weights due to the presence of N-terminal extensions. MUP, 4-methylumbelliferyl phosphate; PRL, phosphatases of regenerating liver.

Human PRLs are characterized by the long half-life of the phosphocysteine intermediate that arises from substrate dephosphorylation. The longevity of the intermediate is attributed to the absence of a serine or threonine residue present in other phosphatases that promotes phosphocysteine hydrolysis (10). To determine whether the formation of a stable phosphocysteine is a property shared by all PRL species, we used Phos-tag SDS-PAGE gels to detect the phosphorylated form (Fig. 1). The PRL proteins were incubated with and without a synthetic substrate and analyzed with and without boiling. Phosphocysteine is degraded by boiling, which distinguishes it from serine, threonine, and tyrosine phosphorylation.

We observed that all the PRLs except *Galdieria* (red algae) and one *Trypanosoma* isoform displayed a slower migrating band in the substrate-incubated, un-boiled lanes, indicating the presence of a stable phosphocysteine intermediate (Fig. 1). The extent of phosphorylation varied with some species showing two bands and other showing only the unphosphorylated or phosphorylated band. This could reflect differences in the stability of intermediate or result from the presence of catalytically inactive protein due to misfolding or oxidation of the catalytic cysteine.

### Burst kinetics is conserved in most PRLs

**Figure 2:**
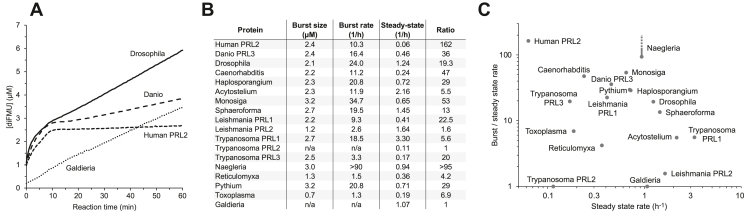
**Burst kinetics is a conserved property of most PRLs.***A*, phosphatase assays with a fluorescent substrate, DiFMUP (25 μM), and recombinant PRLs (3 μM) show burst kinetics for PRLs from animals but not red algae. The burst size is calculated by extrapolating the steady-state rate back to time zero. *B*, table of burst size, burst (initial), and steady-state hydrolysis rates for all PRLs tested. *Naegleria* PRL was the most active enzyme and only a lower bound for the burst rate could be determined. *C*, plot of the burst-like behavior (ratio of initial and steady-state rates) *versus* steady-state rate. All the enzymes, except *Trypanosoma* PRL2 and *Galdieria*, showed some degree of burst kinetics. DiFMUP, 6,8-difluoro-4-methylumbelliferyl phosphate; PRL, phosphatases of regenerating liver.

We next performed dephosphorylation assays to measure the catalytic activity of the different PRL proteins. The assays used a synthetic, fluorogenic substrate and measured the formation of fluorescent product over 1 h (Fig. 2). PRLs from most organisms showed biphasic kinetics with a fast initial burst of activity followed by a slower steady-state rate. The kinetic curves were fit, as described by Bender *et al.* (27), to extract the size of the initial burst, the initial rate, and the steady-state rate.

Four representative traces are shown in Figure 2*A*. PRL from the red algae, *Galdieria*, was one of the only two enzymes that showed a constant, linear increase of product without burst kinetics. PRLs from human, fish, and fly showed an initial burst of product, followed by a slower steady-state hydrolysis. The size of the burst was between 2 and 3 μM, which reflects the generation of cysteine-phosphorylated enzyme during the burst. The steady-state enzyme rates varied from approximately one turnover per hour for the *Drosophila* and *Galdieria* enzymes to only 0.06 for human PRL2.

Figure 2, *B* and *C* show the results of the phosphatase assays with the panel PRL proteins. We used the ratio of the initial and steady-state rates as a measure of the burst-like behavior. Human PRL2 and *Naegleria* showed the largest ratios with initial rates roughly 100-fold faster than the steady-state rates. In contrast, *Galdieria* and *Trypanosoma* PRL2 had ratios of one corresponding to the absence of a burst phase. This is consistent with the absence of a phosphocysteine intermediate on Phos-tag gels (Fig. 1). For most of the samples, the size of the burst was between 2 and 3 μM, which is consistent with the enzyme concentration calculated from the UV absorbance.

### PRLs do not trimerize in solution

**Figure 3:**
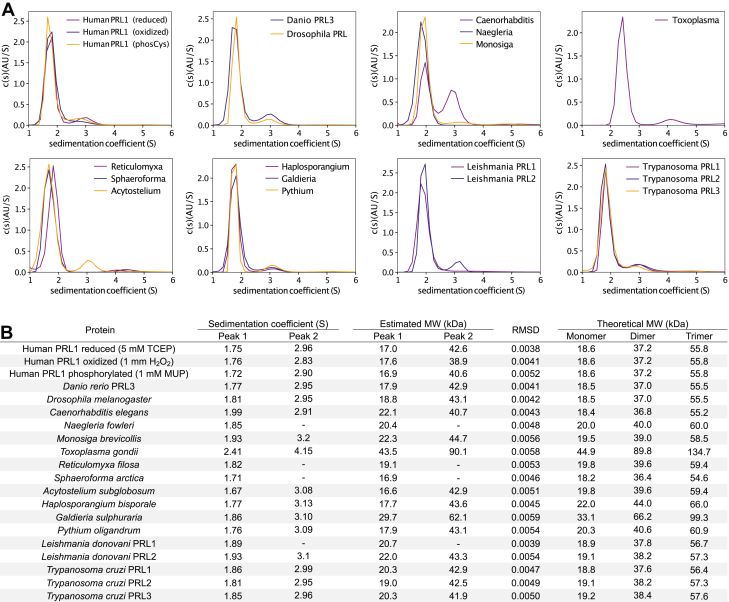
**Self-association of PRLs.***A*, SV-AUC distribution curves for PRL constructs from model organisms. Samples (∼50 μM protein) were analyzed under reducing conditions (5 mM TCEP) except as indicated. *B*, table of sedimentation coefficients and estimated molecular weights. Fitting residuals are shown in Fig. S3. PRL, phosphatases of regenerating liver; SV-AUC, sedimentation velocity analytical ultracentrifugation.

Human PRL1 has been suggested to form trimers in cells based on the observation of trimeric assemblies in crystals and the detection of dimers and trimers following crosslinking (22, 23). To test if nonhuman PRLs form trimers, we analyzed the proteins by sedimentation velocity analytical ultracentrifugation (Figs. 3 and S2). At ∼50 μM concentrations, all the proteins sedimented as monomers with a few constructs displaying minor amounts of dimeric self-association. *Caenorhabditis elegans* PRL displayed the largest amount of a dimer species. As oxidation or phosphorylation of the catalytic cysteine might have affected self-association, we also analyzed human PRL1 in the presence of 1 mM H_2_O_2_ to form the disulfide or 1 mM 4-methylumbelliferyl phosphate (MUP) to generate the phosphocysteine intermediate but did not detect any evidence of trimers. It remains conceivable that prenylated PRLs might trimerize inside cells, but the soluble, unmodified forms do not.

### CNNM binding is a conserved property of PRLs

**Figure 4:**
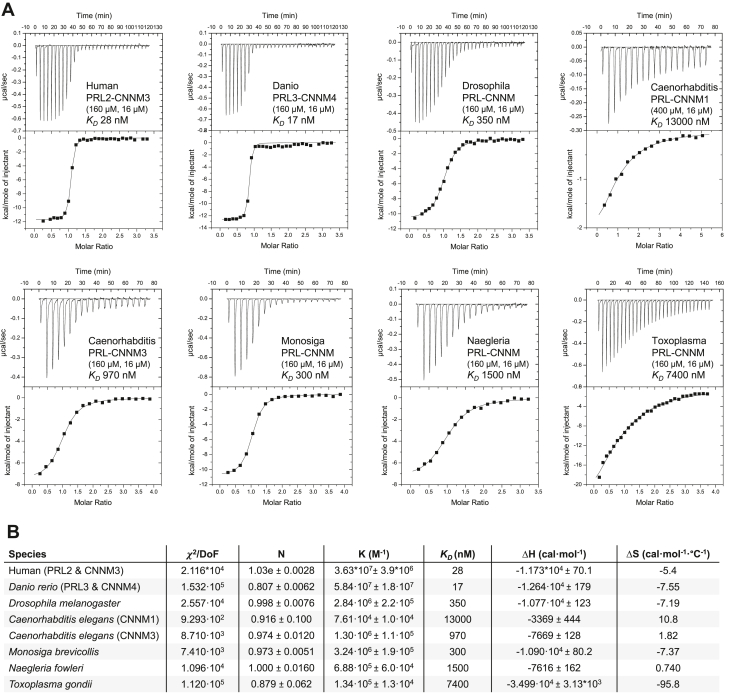
**ITC profiles of CBS-PRL pairs from model organisms.***A*, thermograms and integrated heats of binding for CBS-PRL pairs with calculated binding affinities (*K*_*D*_). *B*, fitting parameters for the thermograms. CBS, cystathionine-β-synthase; ITC, isothermal titration calorimetry; PRL, phosphatases of regenerating liver.

We next sought to determine if the PRL–CNNM interaction is conserved throughout evolution. Isothermal titration calorimetry (ITC) experiments were performed between the PRL and CBS-pair domains of eight selected organisms (Fig. 4 and Table S2). Binding was detected in all cases with affinities ranging from mid-nanomolar for vertebrates to low micromolar for the parasite *Toxoplasma gondi* and the amoeba *Naegleria fowleri*. Although *C. elegans* has only one PRL gene, there are five predicted CNNM proteins. We were able to express the CBS-pair domains for two of these, which bound the *C. elegans* PRL with 1 μM and 13 μM affinities. The *Drosophila* CNNM protein, encoded by the *uex* gene, bound *Drosophila* PRL with 350 nM affinity.

### Crystal structure of the Drosophila PRL–CBS-pair complex

**Figure 5:**
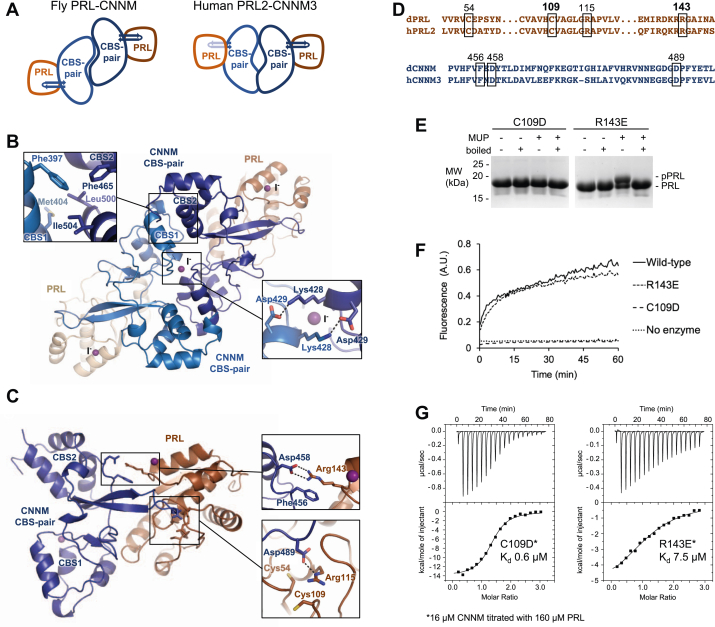
**Structure of *Drosophila* PRL bound to the CBS-pair domain of *Drosophila* CNNM.***A*, comparison of CBS-pair dimerization. The fly CBS-pair domains form an antiparallel dimer, unlike the parallel arrangement of CBS-pair dimers from mammalian CNNM proteins. An extended β-sheet from each CBS-pair monomer binds one PRL protein. *B*, protein-protein contacts between the fly CBS-pair domains. The *inset* show hydrophobic interface and polar contacts around one of the three iodide ions (I^−^) in the crystal. *C*, contacts between the CBS-pair domain and PRL. Binding is mediated by an extended β-sheet that inserts CNNM aspartic acid residue 458 into the PRL active site formed by cysteine 109 and arginine 115. The catalytic cysteine 109 and the regulatory cysteine 54 are both reduced. An intermolecular salt bridge between a CNNM aspartic acid 458 and PRL arginine 143 is conserved between the human and fly complexes. The interaction is stabilized by the stacking of phenylalanine 456 with the arginine side-chain. *D*, conservation of key residues between the *Drosophila* (dPRL, dCNNM) and human (hPRL2, hCNNM3) proteins. *E*, Phos-tag SDS-PAGE analysis showing the formation of phosphocysteine intermediate by the R143E mutant but not C109D in the presence of the substrate MUP. *F*, phosphatase assays with DiFMUP showing WT activity by the *Drosophila* PRL R143E mutant but no activity for C109D. *G*, ITC analysis of complex formation shows the C109D mutation diminishes binding affinity less than two-fold, while the R143E mutation weakens it 20-fold. CBS, cystathionine-β-synthase; DiFMUP, 6,8-difluoro-4-methylumbelliferyl phosphate; MUP, 4-methylumbelliferyl phosphate; PRL, phosphatases of regenerating liver.

To gain a greater understanding of the *Drosophila* PRL–CNNM complex, we carried out structural analysis using X-ray crystallography. A truncated PRL construct (residues 10–161) with surface-exposed cysteine mutated to alanine (C104A) was added to the CNNM CBS-pair and the complex purified by size-exclusion chromatography. The complex was crystallized, and a diffraction dataset obtained at 2.5 Å resolution in a P4_1_2_1_2 space group (Table S3). The crystal structure was solved by molecular replacement using the structure of human complex of PRL2 and the CBS-pair of CNNM3 (PDB entry 5K22) (13). The asymmetric unit of the crystal contained two copies of PRL–CBS complexes in an unusual antiparallel orientation (Fig. 5 and Fig. S3).

CBS-pair domains are a common structural fold and often associate as dimers with ATP bound (28). The CBS-pair domain itself is composed of two sequential CBS motifs that fold together as a unit. Previous structures of CNNM CBS-pairs generally show the domains as symmetric dimers with a parallel head-to-head alignment; however, exceptions exist (13, 16, 24, 29, 30, 31, 32). Uniquely, in the fly complex, the CBS-pair domains crystallized in a head-to-tail orientation (Figs. 5*A* and S3*A*). This leads to novel interdomain contacts with the CBS1 motif of one domain opposing the CBS2 motif of the other (Fig. 5*B*). Dimerization is stabilized by hydrophobic contacts between Phe397, Met404, Phe465, Leu500, and Ile504 along with polar interactions such as between Lys428 and Asp249 at the center of the dimer. While novel for CNNM proteins, it is not clear whether the head-to-tail dimerization property is an intrinsic property of the *Drosophila* CNNM protein. CBS-pair domains show considerable conformational variability, and antiparallel dimers have been observed in other proteins (28). Interestingly, a sequence alignment of the CBS-pair domains of *Drosophila* CNNM and human CNNM3 reveals a conservation of residues involved in both parallel and antiparallel dimerization (Fig. S4), suggesting that both orthologs may exist in either rearrangement in solution. It is possible that the antiparallel orientation arises from crystal packing forces or the presence of iodide in the crystallization buffer.

Three iodide ions from the crystallization buffer are present in the crystal asymmetric unit. Iodides are often found near arginine and lysine residues in hydrophobic/positively charged cavities and have been used for single wavelength anomalous dispersion phasing (33). We were able to use the anomalous signals to confirm the iodide positions (Fig. S3*B*). One iodide ion is stabilized by Lys428 and Tyr426 at the dimerization interface of the CBS-pair domains, whereas the two other iodides bind at the interface between PRL (Arg139, Arg143) and a neighboring CBS-pair from a symmetry mate (Arg372, Lys373).

The fly PRL and CBS-pair domains themselves are quite similar to the human proteins. Structural alignments give a RMSD of 1.1 Å for PRLs and 1.9 Å for the CBS-pair domains. The contacts between the domains also match those observed with the human proteins. CNNM interacts through an extended loop that inserts into the active site of PRL (Fig. 5*C*). CNNM Asp489 acts as a pseudosubstrate and mimics the negative charge of a phosphorylated substrate interacting with active site residues Arg115 and Cys109. The catalytic cysteine of fly PRL (Cys109) is adjacent to a regulatory cysteine (Cys54). In human PRLs, the cysteines form an intramolecular disulfide bond under oxidizing or even mildly reducing conditions. In fly protein, both Cys54 and Cys109 are found in the reduced state (Figs. 5*C* and S3*A*). Outside of the catalytic site, CNNM Asp458 makes polar contacts with PRL Arg143 as observed in structures of the human proteins. The guanidinium side-chain is aligned with the side-chain of Phe456 in a typical planar stacking interaction (34).

Overlaying the human and *Drosophila* complexes reveals a small shift in the angle between the two proteins which generates an additional contact surface between the first PRL α-helix and CNNM residues from the first CBS motif (Fig. S5). This contact does not appear to contribute significantly to the stability of the complex since the affinity of the fly complex is 20-fold weaker than the human complex. Most likely, the differences between the fly and human structures are the result of crystal packing forces.

### Mutagenesis of Drosophila PRL–CBS-pair complex

PRL and CNNM function together to protect neurons in flies but it is not known if this is due to complex formation or PRL phosphatase activity (35). Based on studies of human PRLs, we generated mutations in fly PRL to selectively inactivate either CNNM binding or phosphatase activity (Fig. 5*D*). In human PRL3, substitution of the catalytic cysteine by aspartic acid inactivates phosphatase activity and preserves CNNM binding, while loss of an arginine residue at the intermolecular interaction has the reciprocal effect (14). The corresponding mutations, C109D and R143E, in *Drosophila* PRL were prepared and tested for their ability to form the phosphocysteine intermediate, dephosphorylate 6,8-difluoro-4-methylumbelliferyl phosphate, and bind CNNM (Fig. 5, *E*–*G*).

Phos-tag gels confirmed that the C109D mutation prevented formation of the phosphocysteine intermediate, while the R143E mutation allowed formation of the phosphocysteine intermediate (Fig. 5*E*). The catalytic effects of the mutations were confirmed by phosphatase assays: C109D mutant was inactive, while the R143E mutant displayed WT activity (Fig. 5*F*). Finally, ITC experiments showed the opposite behavior with close to WT CNNM-binding affinity for the C109D mutant and a 20-fold drop in affinity for the R143E mutant (Fig. 5*G*).

## Discussion

PRLs are widely conserved from worms to flies, and developmental roles have been characterized in several animal model systems (36). Zebrafish has five PRL isoforms. Morpholino-mediated inhibition of the most highly expressed forms causes defects in development and apoptosis (37). The PRL3a isoform was found to control melanocyte stem cell expansion and differentiation (38). In flies, the single PRL isoform was shown to play roles in the nervous system, directing synaptogenesis and protecting against CO_2_-dependent overstimulation, as well as wing vein development (35, 39, 40). The fly CNNM homolog, encoded by the *unextended* (*uex*) gene, has been implicated in long-term memory development and circadian rhythms (41, 42). In nematodes, PRL was shown to be required for proper trafficking of lysosomal proteins (43). PRLs are also found in parasites where they play roles in virulence and survival (44, 45, 46).

Despite their broad distribution and importance in cancer and development, PRLs are poorly understood (15). They are highly potent oncogenes implicated in tumorigenesis and metastasis but have no confirmed substrates. *In vitro*, they have very low levels of catalytic activity but are largely present *in vivo* in a cysteine-phosphorylated intermediate state, which is a direct result of catalytic activity. They regulate CNNM proteins by binding as pseudophosphatases, but the binding is regulated by their catalytic activity. Their phosphatase activity is highly conserved but not required for the regulation of CNNM proteins or PRL oncogenicity in a model of metastasis (14).

**Figure 6:**
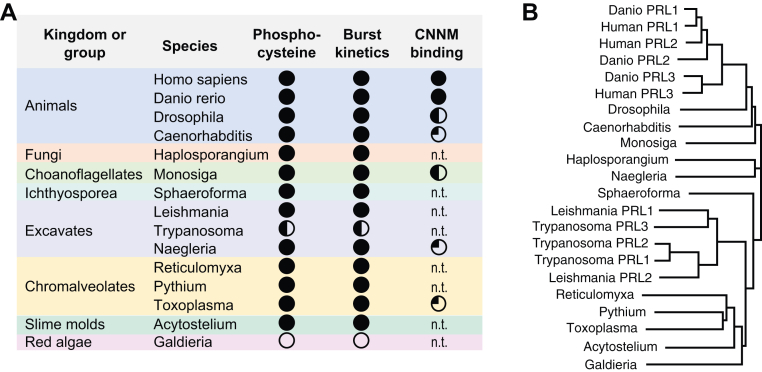
**Conservation of PRL function.** Comparison of (*A*) properties of PRL proteins in *in vitro* assays with their (*B*) phylogeny. Phosphocysteine (*filled circles*) was detected for all PRLs except *Galdieria* and one *Trypanosoma* isoform. Phosphatase assays showed burst kinetics (*filled circles*) except for *Galdieria* and one *Trypanosoma* isoform. CNNM binding affinities ranged from low nanomolar (*filled circles*) to high nanomolar (*half-filled circles*) and low micromolar (*quarter-filled*). n.t. = not tested. Phylogeny tree was calculated by neighbor joining the amino acid sequences. ITC, isothermal titration calorimetry; PRL, phosphatases of regenerating liver.

To gain insight into the seemingly conflicting roles of PRL catalysis and CNNM binding, we have characterized the *in vitro* properties of PRL proteins from a diverse set of eukaryotes. We found that both the unusual catalytic properties of PRLs and their ability to bind CNNM proteins are highly conserved (Fig. 6). All the PRL proteins showed steady-state hydrolysis rates *in vitro* that are a thousand-fold slower than those of structurally similar phosphatases, such as CDC14 (47, 48). Except for red algae and some parasitic forms, all the PRLs showed burst kinetic behavior and the accumulation of phosphocysteine in the presence of substrate. The slow kinetics cannot be attributed to the absence of the proper substrate in the *in vitro* assays since the rate limiting step is the second step in the catalytic cycle. The step is substrate independent and only depends on the rate of hydrolysis of the phosphocysteine intermediate (15).

A conserved structural feature of PRLs is the substitution of a serine or threonine residue in the catalytic site by alanine (Ala111 in human PRL3). Reversion of this amino acid to the hydroxyl-bearing amino acid found in other protein phosphatases increases the steady state activity and eliminates the burst effect (10). A related mechanism may be responsible for the absence of burst kinetics for *Galdieria* and *Trypanosoma* PRL2 as they are only proteins with a serine or threonine close to the P-loop catalytic cysteine (Fig. S1). It is notable that the *Trypanosoma* PRL2 and PRL1 catalytic domains are 95% identical with the alanine-to-serine substitution being the only significant difference in the sequences of catalytic sites.

The parallel conservation of burst-like kinetics and CNNM binding suggests that both play a role in PRL signaling. Although ITC experiments measured a wide range of CNNM-binding affinities, even micromolar affinity is likely to be physiologically relevant for the interaction of proteins on a two-dimensional membrane. All the PRLs contain a CAAX box and are likely prenylated. The structure of the PRL–CNNM complex from flies confirms the interaction surfaces are conserved and additionally suggests that phosphorylation of the *Drosophila* PRL catalytic cysteine will block CNNM binding, as observed with the human proteins (13).

These findings open the door to studying PRL functions in experimentally tractable organisms with well-developed genetic and analytic tools. The identification of PRL mutations that differentially affect phosphatase activity and CNNM binding will allow future studies to test if the neurodevelopmental and protective functions in flies act through CNNM or still unidentified substrates. Understanding the function of PRLs in these phylogenetically diverse organisms promises novel insights into human diseases.

## Experimental procedures

### Cloning of DNA constructs

Coding DNAs were codon-optimized for *Escherichia coli* expression and chemically synthesized (Bio Basic Inc). PRLs without the C-terminal prenylation site were cloned into NdeI and BamHI sites of pET15b(+) vector (Novagen Inc) with an N-terminal His_6_-tag (Table S2). CNNM CBS-pair domains were cloned into BamHI and XhoI sites of pGEX-6P-1 vector (Amersham-Pharmacia) with an N-terminal GST-tag (Table S2). Plasmids expressing PRL proteins from *Leishmania* and *Trypanosoma* were obtained from the Seattle Structural Genomics Center for Infectious Disease (49). For crystallization, residues 14 to 165 of *Drosophila melanogaster* PRL were subcloned into the NdeI and XhoI sites of pET29a vector (Millipore Sigma) with a C-terminal His_6_-tag. Mutations of *D. melanogaster* PRLs (C104A, C109D, R143E) were performed using the Quick-Change Lightning Site-Directed Mutagenesis Kit (Agilent). The plasmids expressing human PRL2 and CNNM3 CBS-pair domain were described previously (13). All constructs were verified by Sanger sequencing and transformed into *E. coli* strain BL21(DE3).

### Expression and purification of recombinant proteins

Cultures were grown at 37 °C in LB medium to an optical density of 0.8 and induced overnight with 1 mM IPTG at 15 °C. Cells were pelleted the following day by centrifuging at 5000*g* for 20 min at 4 °C. The pellet was resuspended in lysis buffer (50 mM Hepes pH 7.5, 500 mM NaCl, 5% glycerol, 10 mM β-mercaptoethanol) and lysed by sonication. Cellular debris was removed by centrifugation at 50,000*g* for 30 min at 4 °C, and the supernatant was loaded onto the appropriate resin for affinity purification.

GST-tagged CNNM CBS-pair domains were incubated in glutathione-Sepharose resin (GE Healthcare), washed with lysis buffer, and eluted with lysis buffer containing 20 mM of reduced glutathione. For crystallization, affinity purification of the CBS-pair domain of *D. melanogaster* CNNM was followed by overnight 3C protease cleavage at 4 °C to remove the GST tag, leaving an N-terminal Gly-Pro-Leu-Gly-Ser (GPLGS) extension. His_6_-tagged PRLs were incubated in TALON Co^2+^ affinity resin (Takara Bio), washed with lysis buffer containing 25 mM imidazole, and eluted with lysis buffer containing 500 mM of imidazole. Proteins were further purified by a Superdex-75 size-exclusion chromatography column (GE Healthcare) in HPLC buffer (20 mM Hepes pH 7.5, 100 mM NaCl, 5 mM TCEP).

To prepare the *D. melanogaster* PRL/CBS-pair complex for crystallization, HPLC-purified *D. melanogaster* PRL (res. 14–165) C104A and GPLGS-CBS-pair were mixed in a 2:1 M ratio and incubated for 30 min at 4 °C. The complex was then purified from the unbound proteins by a Superdex-200 size-exclusion chromatography column (GE Healthcare) in HPLC buffer.

Purified proteins were verified by SDS-PAGE analysis, and concentrations were determined using UV absorbance at 280 nm and theoretical extinction coefficients.

### Detection of substrate-mediated PRL phosphorylation

Fifty micromolars of purified PRLs, suspended in HPLC buffer, were incubated with or without 1 mM MUP for 60 min at 37 °C. To detect the formation of phosphocysteine intermediates in PRLs, samples were resolved on 12.5% Tris-glycine gels polymerized with 20 μM Phos-tag reagent (Cedarlane) and 40 μM ZnCl_2_. Dephosphorylated PRLs were generated by boiling samples in SDS-PAGE loading buffer for 10 min at 90 °C.

### Isothermal titration calorimetry

ITC experiments were performed on a VP-ITC titration calorimeter (MicroCal) at 20 °C in HPLC buffer. GST-CBS-pairs were titrated with PRL using 29 injections (5 μl for the first injection, 10 μl for subsequent injections) for human, *D*. *rerio*, *D. melanogaster* (wild-type), and *T*. *gondii*. Nineteen injections (5 μl for the first injection, followed by 15 μl injections) were performed for *D. melanogaster* mutants (C109D, R143E), *C. elegans*, *M*. *brevicollis*, and *N. fowleri*. Results were analyzed using ORIGIN v7 software (https://www.originlab.com/) and fitted to a single site-binding model. Concentrations used for the titration experiments are indicated in the individual figure panels.

### Dephosphorylation assay

Phosphatase activity was measured by incubating 3 μM of PRL in 50 μl of HPLC buffer supplemented with 25 μM of 6,8-difluoro-4-methylumbelliferyl phosphate (Molecular Probes, Thermo Fisher Scientific) at room temperature for 1 h. Measurements of the fluorescent product, 6,8-difluoro-7-hydroxy-4-methylcoumarin, were taken every 30 s using excitation at 360 nm and emission at 455 nm on a SpectraMax Paradigm plate reader (Molecular Devices, LLC). The fluorescence intensity was converted to molar concentration using standards of 6,8-difluoro-7-hydroxy-4-methylcoumarin. Kinetic parameters were extracted from the activity traces by fitting them as the sum of an exponentially decreasing burst and a linear steady-state rate (27) (Supplemental Excel File).

### Sedimentation velocity analytical ultracentrifugation

Sedimentation velocity analytical ultracentrifugation experiments were performed at 20 °C using a Beckman Coulter XL -I analytical ultracentrifuge using an An-60Ti rotor at 98,000*g* (35,000 RPM) for 18 h with scans performed every 60 s. A double-sector cell, equipped with a 12 mm Epon centerpiece and sapphire windows, was loaded with 380 μl of protein at A_280_ of ∼1 (roughly 50 μM) in HPLC buffer. Human PRL1 was additionally analyzed following exchange into HPLC buffer with 10 mM 1,4-DTT in place of TCEP, 1 mM H_2_O_2_ without TCEP, or 1 mM MUP with 5 mM TCEP. Sedimentation was monitored using absorbance at 280 nm. The data were analyzed with Sedfit v1501b using a continuous c(s) distribution (50). Buffer density and viscosity were determined to be 1.0039 g/cm^3^ and 0.01026 mPa·s with Sednterp (51). Partial specific volumes for PRLs were defaulted to 0.73 cm^3^/g, and the frictional ratio (f/f_0_) was floated. Residual and c(s) distribution graphs were plotted using GUSSI (52).

### Crystallization

Crystals of the PRL–CBS-pair complex from *D. melanogaster* were grown using the sitting-drop vapor-diffusion method by the mixture of 1 μl of protein complex (11 mg/ml) in HPLC buffer with 1 μl of (0.1 M Bis-tris propane pH 6.5, 17% (w/v) PEG3350, 0.2 M NaI) at 4 °C. Crystals appeared within 1 to 2 days. After a week, crystals were cryoprotected in mother liquor supplemented with 25% (v/v) ethylene glycol and frozen in liquid nitrogen.

### Crystal data collection, structure determination, and structure refinement

Diffraction data for the crystal was collected at the CMCF-08B1-1 beamline at the Canadian Light Source. A total of 1440 images were collected with an oscillation angle of 0.25° and wavelength of 0.95374 Å. The reflections were integrated and scaled with HKL-2000 (53). The structures of the PRL and CBS-pair proteins were solved by molecular replacement with the Phaser tool of the PHENIX software package (54) using human PRL2 and CNNM3 (PDB entry 5K22). Model building was performed on Coot (55) and the model refined with the PHENIX refinement tool. Real-space torsion angle refinement, individual B-factors, and noncrystallographic symmetry were used throughout the refinement. Figures were generated with PyMOL (Schrödinger, Inc).

## Data availability

The atomic coordinates and structure factors (code 8CT8) have been deposited in the Protein Data Bank.

## Supporting information

This article contains supporting information (56, 57).

## Conflict of interest

The authors declare that they have no conflicts of interest with the contents of this article.

## References

[1] Diamond R.H., Cressman D.E., Laz T.M., Abrams C.S., Taub R. (1994). PRL-1, a unique nuclear protein tyrosine phosphatase, affects cell growth. Mol. Cell. Biol..

[2] Saha S., Bardelli A., Buckhaults P., Velculescu V.E., Rago C., St Croix B. (2001). A phosphatase associated with metastasis of colorectal cancer. Science.

[3] Hardy S., Kostantin E., Hatzihristidis T., Zolotarov Y., Uetani N., Tremblay M.L. (2018). Physiological and oncogenic roles of the PRL phosphatases. FEBS J..

[4] Frankson R., Yu Z.H., Bai Y., Li Q., Zhang R.Y., Zhang Z.Y. (2017). Therapeutic targeting of oncogenic tyrosine phosphatases. Cancer Res..

[5] Tasker N.R., Rastelli E.J., Burnett J.C., Sharlow E.R., Lazo J.S., Wipf P. (2019). Tapping the therapeutic potential of protein tyrosine phosphatase 4A with small molecule inhibitors. Bioorg. Med. Chem. Lett..

[6] Thura M., Al-Aidaroos A.Q., Gupta A., Chee C.E., Lee S.C., Hui K.M. (2019). PRL3-zumab as an immunotherapy to inhibit tumors expressing PRL3 oncoprotein. Nat. Commun..

[7] Al-Aidaroos A.Q., Zeng Q. (2010). PRL-3 phosphatase and cancer metastasis. J. Cell. Biochem..

[8] Rios P., Li X., Kohn M. (2013). Molecular mechanisms of the PRL phosphatases. FEBS J..

[9] Funato Y., Miki H. (2014). Reversible oxidation of PRL family protein-tyrosine phosphatases. Methods.

[10] Kozlov G., Cheng J., Ziomek E., Banville D., Gehring K., Ekiel I. (2004). Structural insights into molecular function of the metastasis-associated phosphatase PRL-3. J. Biol. Chem..

[11] Guan K.L., Dixon J.E. (1991). Evidence for protein-tyrosine-phosphatase catalysis proceeding via a cysteine-phosphate intermediate. J. Biol. Chem..

[12] Pannifer A.D., Flint A.J., Tonks N.K., Barford D. (1998). Visualization of the cysteinyl-phosphate intermediate of a protein-tyrosine phosphatase by X-ray crystallography. J. Biol. Chem..

[13] Gulerez I., Funato Y., Wu H., Yang M., Kozlov G., Miki H. (2016). Phosphocysteine in the PRL-CNNM pathway mediates magnesium homeostasis. EMBO Rep..

[14] Kozlov G., Funato Y., Chen Y.S., Zhang Z., Illes K., Miki H. (2020). PRL3 pseudophosphatase activity is necessary and sufficient to promote metastatic growth. J. Biol. Chem..

[15] Gehring K., Kozlov G., Yang M., Fakih R. (2022). The double lives of phosphatases of regenerating liver: a structural view of their catalytic and noncatalytic activities. J. Biol. Chem..

[16] Zhang H., Kozlov G., Li X., Wu H., Gulerez I., Gehring K. (2017). PRL3 phosphatase active site is required for binding the putative magnesium transporter CNNM3. Sci. Rep..

[17] Funato Y., Yamazaki D., Mizukami S., Du L., Kikuchi K., Miki H. (2014). Membrane protein CNNM4-dependent Mg^2+^ efflux suppresses tumor progression. J. Clin. Invest..

[18] Hardy S., Uetani N., Wong N., Kostantin E., Labbe D.P., Begin L.R. (2015). The protein tyrosine phosphatase PRL-2 interacts with the magnesium transporter CNNM3 to promote oncogenesis. Oncogene.

[19] Funato Y., Miki H. (2019). Molecular function and biological importance of CNNM family Mg^2+^ transporters. J. Biochem..

[20] Hirata Y., Funato Y., Takano Y., Miki H. (2014). Mg^2+^-dependent interactions of ATP with the cystathionine-beta-synthase (CBS) domains of a magnesium transporter. J. Biol. Chem..

[21] Kim K.A., Song J.S., Jee J., Sheen M.R., Lee C., Lee T.G. (2004). Structure of human PRL-3, the phosphatase associated with cancer metastasis. FEBS Lett..

[22] Jeong D.G., Kim S.J., Kim J.H., Son J.H., Park M.R., Lim S.M. (2005). Trimeric structure of PRL-1 phosphatase reveals an active enzyme conformation and regulation mechanisms. J. Mol. Biol..

[23] Sun J.P., Wang W.Q., Yang H., Liu S., Liang F., Fedorov A.A. (2005). Structure and biochemical properties of PRL-1, a phosphatase implicated in cell growth, differentiation, and tumor invasion. Biochemistry.

[24] Gimenez-Mascarell P., Oyenarte I., Hardy S., Breiderhoff T., Stuiver M., Kostantin E. (2017). Structural basis of the oncogenic interaction of phosphatase PRL-1 with the magnesium transporter CNNM2. J. Biol. Chem..

[25] Denu J.M., Dixon J.E. (1995). A catalytic mechanism for the dual-specific phosphatases. Proc. Natl. Acad. Sci. U. S. A..

[26] Zhang Z.Y., Palfey B.A., Wu L., Zhao Y. (1995). Catalytic function of the conserved hydroxyl group in the protein tyrosine phosphatase signature motif. Biochemistry.

[27] Bender M.L., Kezdy F.J., Wedler F.C. (1967). Alpha-chymotrypsin: enzyme concentration and kinetics. J. Chem. Educ..

[28] Ereno-Orbea J., Oyenarte I., Martinez-Cruz L.A. (2013). CBS domains: ligand binding sites and conformational variability. Arch. Biochem. Biophys..

[29] Chen Y.S., Kozlov G., Fakih R., Funato Y., Miki H., Gehring K. (2018). The cyclic nucleotide–binding homology domain of the integral membrane protein CNNM mediates dimerization and is required for Mg^2+^ efflux activity. J. Biol. Chem..

[30] Chen Y.S., Kozlov G., Fakih R., Yang M., Zhang Z., Kovrigin E.L. (2020). Mg(2+)-ATP sensing in CNNM, a putative magnesium transporter. Structure.

[31] Gimenez-Mascarell P., Gonzalez-Recio I., Fernandez-Rodriguez C., Oyenarte I., Muller D., Martinez-Chantar M.L. (2019). Current structural knowledge on the CNNM family of magnesium transport mediators. Int. J. Mol. Sci..

[32] Chen Y.S., Kozlov G., Moeller B.E., Rohaim A., Fakih R., Roux B. (2021). Crystal structure of an archaeal CorB magnesium transporter. Nat. Commun..

[33] Abendroth J., Gardberg A.S., Robinson J.I., Christensen J.S., Staker B.L., Myler P.J. (2011). SAD phasing using iodide ions in a high-throughput structural genomics environment. J. Struct. Funct. Genomics.

[34] Flocco M.M., Mowbray S.L. (1994). Planar stacking interactions of arginine and aromatic side-chains in proteins. J. Mol. Biol..

[35] Guo P., Xu X., Wang F., Yuan X., Tu Y., Zhang B. (2019). A novel neuroprotective role of phosphatase of regenerating liver-1 against CO2 stimulation in Drosophila. iScience.

[36] Lin M.D., Lee H.T., Wang S.C., Li H.R., Hsien H.L., Cheng K.W. (2013). Expression of phosphatase of regenerating liver family genes during embryogenesis: an evolutionary developmental analysis among drosophila, amphioxus, and zebrafish. BMC Dev. Biol..

[37] Lohani S., Funato Y., Akieda Y., Mizutani K., Takai Y., Ishitani T. (2022). A novel role for PRL in regulating epithelial cell density by inducing apoptosis at confluence. J. Cell Sci..

[38] Johansson J.A., Marie K.L., Lu Y., Brombin A., Santoriello C., Zeng Z. (2020). PRL3-DDX21 transcriptional control of endolysosomal genes restricts melanocyte stem cell differentiation. Dev. Cell.

[39] Urwyler O., Izadifar A., Vandenbogaerde S., Sachse S., Misbaer A., Schmucker D. (2019). Branch-restricted localization of phosphatase Prl-1 specifies axonal synaptogenesis domains. Science.

[40] Zheng H., Lou Z., Yuan X., Wu H., Yang X., Xi Y. (2022). Phosphatase of regenerating liver-1 regulates wing vein formation through TGF-beta pathway in Drosophila melanogaster. Front. Biosci. (Landmark Ed.).

[41] Wu Y., Funato Y., Meschi E., Jovanoski K.D., Miki H., Waddell S. (2020). Magnesium efflux from Drosophila Kenyon cells is critical for normal and diet-enhanced long-term memory. Elife.

[42] Kula-Eversole E., Lee D.H., Samba I., Yildirim E., Levine D.C., Hong H.K. (2021). Phosphatase of regenerating liver-1 selectively times circadian behavior in darkness via function in PDF neurons and dephosphorylation of TIMELESS. Curr. Biol..

[43] Funato Y., Yoshida A., Hirata Y., Hashizume O., Yamazaki D., Miki H. (2020). The oncogenic PRL protein causes acid addiction of cells by stimulating lysosomal exocytosis. Dev. Cell.

[44] Yang C., Blakely W.J., Arrizabalaga G. (2022). The tyrosine phosphatase PRL regulates attachment of toxoplasma gondii to host cells and is essential for virulence. mSphere.

[45] Leitherer S., Clos J., Liebler-Tenorio E.M., Schleicher U., Bogdan C., Soulat D. (2017). Characterization of the protein tyrosine phosphatase LmPRL-1 secreted by Leishmania major via the exosome pathway. Infect. Immun..

[46] Cuevas I.C., Rohloff P., Sanchez D.O., Docampo R. (2005). Characterization of farnesylated protein tyrosine phosphatase TcPRL-1 from Trypanosoma cruzi. Eukaryot. Cell.

[47] Gray C.H., Good V.M., Tonks N.K., Barford D. (2003). The structure of the cell cycle protein Cdc14 reveals a proline-directed protein phosphatase. EMBO J..

[48] Wang W.Q., Bembenek J., Gee K.R., Yu H., Charbonneau H., Zhang Z.Y. (2004). Kinetic and mechanistic studies of a cell cycle protein phosphatase Cdc14. J. Biol. Chem..

[49] Myler P.J., Stacy R., Stewart L., Staker B.L., Van Voorhis W.C., Varani G. (2009). The Seattle structural genomics center for infectious disease (SSGCID). Infect. Disord. Drug Targets.

[50] Schuck P. (2004). A model for sedimentation in inhomogeneous media. I. Dynamic density gradients from sedimenting co-solutes. Biophys. Chem..

[51] Laue T.M., Shah B.D., Ridgeway T.M., Pelletier S.L., Harding S., Rowe A., Horton J. (1992). Analytical Ultracentrifugation in Biochemistry and Polymer Science.

[52] Brautigam C.A. (2015). Calculations and publication-quality illustrations for analytical ultracentrifugation data. Methods Enzymol..

[53] Lor L.A., Schneck J., McNulty D.E., Diaz E., Brandt M., Thrall S.H. (2007). A simple assay for detection of small-molecule redox activity. J. Biomol. Screen..

[54] Adams P.D., Afonine P.V., Bunkoczi G., Chen V.B., Davis I.W., Echols N. (2010). Phenix: a comprehensive python-based system for macromolecular structure solution. Acta Crystallogr. D Biol. Crystallogr..

[55] Emsley P., Cowtan K. (2004). Coot: model-building tools for molecular graphics. Acta Crystallogr. D Biol. Crystallogr..

[56] Waterhouse A.M., Procter J.B., Martin D.M.A., Clamp M., Barton G.J. (2009). Jalview Version 2—a multiple sequence alignment editor and analysis workbench. Bioinformatics.

[57] Sievers F., Higgins D.G. (2018). Clustal Omega for making accurate alignments of many protein sequences. Protein Sci.

