# Supplementary material for: Burst kinetics and CNNM binding are evolutionarily conserved properties of phosphatases of regenerating liver
Source: J Biol Chem. 2023 Feb 22;299(4):103055. doi: 10.1016/j.jbc.2023.103055 (PMC10040874; doi:10.1016/j.jbc.2023.103055)
Supplement: Supporting Figures S1–S5 and Tables S1–S3 [file mmc2.pdf]

Supplemental Information for

Burst kinetics and CNNM binding are evolutionarily conserved properties of phosphatases of regenerating liver (PRL)

Rayan Fakih, Robert H. Goldstein, Guennadi Kozlov, Kalle Gehring

Department of Biochemistry & Centre for Structural Biology, McGill University, Montreal, QC H3G 0B1, Canada

<u>Table of Contents</u>

Table S1: Genetic and physiochemical properties of PRLs page 2

Table S2: Summary of proteins constructs studied page 3

Table S3: Crystal structure data collection and refinement statistics page 4

Figure S1. Sequence alignment and key features of PRLs page 5

Figure S2. Analytical ultracentrifugation of PRL proteins page 6

Figure S3. Electron density map of fly PRL/CBS crystal structure elements page 7

Figure S4. Head-to-head dimerization of the CBS-pair domain in human CNNM3 page 8

Figure S5. Comparison of fly and human PRL/CBS-pair structures page 9

Literature cited page 9

Table S1: Genetic and physiochemical properties of PRLs

Super-group	Kingdom	Genus	Paralog	GenBank ID	Identity to hPRL-3 (%)	MW (Da)	Length (# a.a.)	pI
Amorphea (Unikonts)	Animalia	*Homo*	PRL3	NP_116000.1	100	19,535	173	9.3
			PRL2	NP_001356788.1	78	19,127	167	8.7
			PRL1	NP_001372183.1	79	19,815	173	9.2
		Drosophila		NP_001260487.1	56	19,973	176	8.8
		Danio	PRL3	NP_998346.1	87	19,857	173	9.4
			PRL2	NP_001257469.1	68	19,969	174	8.8
			PRL1	NP_001007776.2	76	20,003	173	9.3
		Caenorhabditis		NP_001379679.1	51	21,158	190	9.0
	Choanoflagellatea	*Monosiga*		XP_001747604.1	50	18,240	161	8.3
	Ichthyosporea	*Sphaeroforma*		XP_014159498.1	50	17,074	154	8.3
	Fungi	*Haplosporangium*		KAF8976903.1	44	20,714	185	9.3
	Amoebozoa	*Acytostelium*		XP_012751436.1	46	18,506	166	8.7
Chromalveolates	Foraminifera	*Reticulomyxa*		ETO21150.1	41	18,508	164	8.3
	Alveolata	*Toxoplasma*		KYF41327.1	42	44,052	404	9.3
	Heterokonta	*Pythium*		TMW57706.1	46	19,042	169	7.1
Excavates	Euglenozoa	*Leishmania*	PRL2	XP_001682103.1	40	19534	176	7.7
			PRL1	XP_001682101.1	42	19376	175	8.6
		Trypanosoma	PRL3	XP_805262.1	45	19643	180	8.6
			PRL2	XP_816679.1	38	19533	176	8.3
			PRL1	XP_805801.1	40	19237	176	8.3
	Heterolobosea	*Naegleria*		XP_044560670.1	46	18,747	167	8.6
Plants	Rhodophyta	*Galdieria*		XP_005707647.1	41	31,873	287	9.5

Table S2: Summary of recombinant proteins constructs

Organism	PRL			CNNM CBS-pair domain		
	Residues	Yield[1] (mg/L)	AddGene / SSGCID ID/UniProt ID	Residues	Yield (mg/L)	AddGene ID/UniProt ID
Homo sapiens	9-160 PRL2 (C95A, C96A, C119A)	-	Q12974	299-452 CNNM3	-	Q8NE01
Caenorhabditis elegans	1-186	4.3	166416	376-530 CNNM1	2.5	166508
				326-514 CNNM2	0	166433
				375-527 CNNM3	2.2	166510
				300-461CNNM4	0	166511
				301-476 CNNM5	0	166434
Drosophila melanogaster	1-172	6.1	166418	361-515	4.3	166436
Danio rerio	1-169 PRL3	19.8	166417	347-499 CNNM4	5.4	170785
Toxoplasma gondii	1-395	8.7	170789	193-346	3.9	166438
Naegleria fowleri	1-163	14.2	166421	415-568	5.0	166437
Monosiga brevicollis	1-157	20.8	166420	311-463	4.1	166435
Pythium oligandrum	1-165	14.7	166423			
Reticulomyxa filosa	1-160	25.6	166424			
Acytostelium subglobosum	1-162	15.4	166415			
Haplosporangium bisporale	1-181	2.8	170788			
Sphaeroforma arctica	1-149	16.2	166425			
Galdieria sulphuraria	1-283	4.7	166419			
Leishmania major[2]	1-171 PRL1	5.0	LemaA.20429.a			
	1-172 PRL2	6.0	LemaA.20427.a			
Trypanosoma cruzi[2]	1-172 PRL1	4.2	TrcrB.20427.a			
	1-172 PRL2	2.0	TrcrB.20427.b			
	1-176 PRL3	2.7	TrcrB.20429.a			

[1]Yield of purified protein (mg) per liter of bacterial culture
[2]Plasmids from the Seattle Structural Genomics Center for Infectious Disease (SSGCID)

Table S3: Crystal structure data collection and refinement statistics

**Data collection**	
Wavelength (Å)	0.95374
Space group	$P4_12_12$
**Cell dimensions**	
a, b, c (Å)	83.31, 83.31, 238.19
α, β, γ (°)	90, 90, 90
Resolution (Å)	48.44-2.50 (2.53-2.50)
Redundancy	1.8 (1.9)
R_{merge}	0.042 (0.284)
Completeness (%)	99.72 (99.52)
I/σ(I)	20.04 (2.19)
$CC_{1/2}$	0.995 (0.855)
**Refinement**	
Resolution (Å)	2.50 (2.59)
No. of unique reflections	29936 (2909)
R_{work}/R_{free}	0.225 / 0.275
**No. of atoms**	
Protein	4799
Iodine	3
Waters	54
**B factors**	
Protein	64.88
Iodine	47.58
**RMSDs**	
Bond lengths (Å)	0.002
Bond angles (°)	0.432
**Ramachandran plots**	
Favored (%)	96.55
Allowed (%)	3.45
Disallowed	0.00
**PDB code**	8CT8

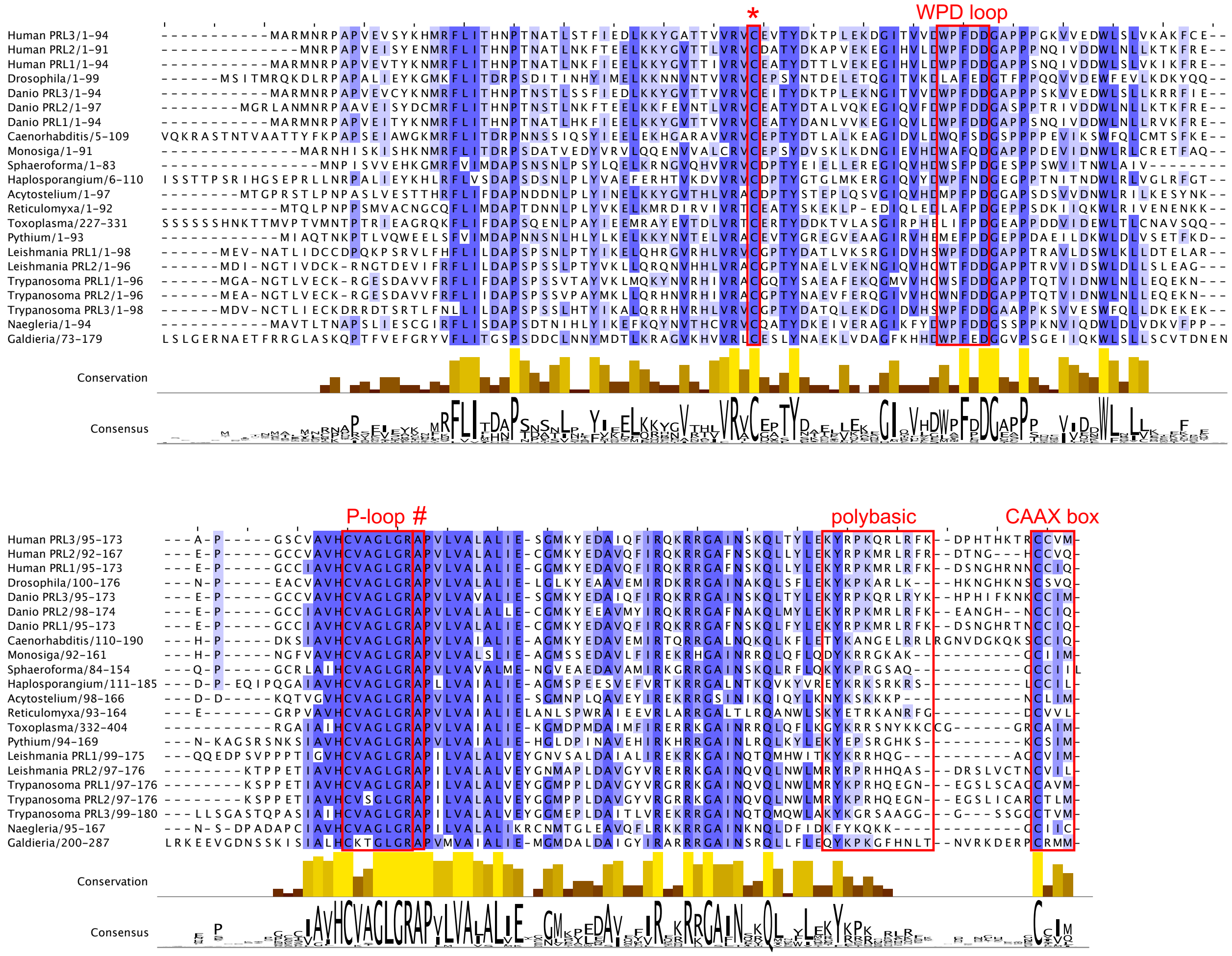

Figure S1. Sequence alignment and key features of PRLs. Alignment was computed in Clustal Omega and presented in Jalview (1,2). Residues are colored by sequence similarity with darker shades of blue meaning higher similarity. Key motifs and residues are boxed in red. The conserved cysteine that can form a disulfide with the P-loop catalytic cysteine is identified by an asterisk (*). The alanine in the P-loop that is responsible for the long lifetime of the phosphocysteine intermediate is marked by a pound sign (#). The absence of burst kinetics for Galdieria and Trypansoma PRL2 may be related to the presence of a threonine or serine in their P-loops. Following a polybasic region, PRLs are prenylated on the cysteine in the C-terminal CAAX box. Portions of the *Toxoplasma* and *Galdieria* sequences are not shown.

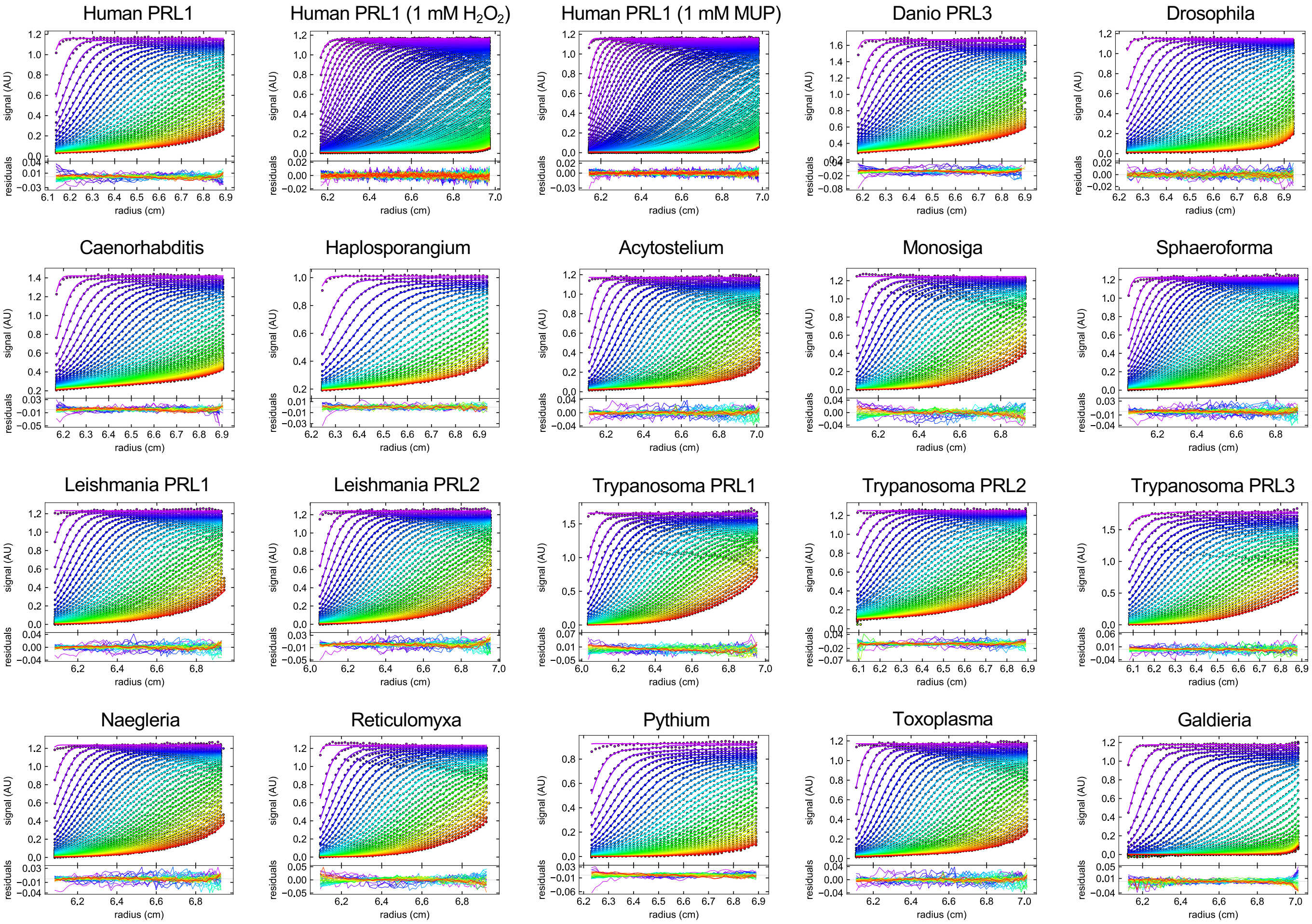

Figure S2. Analytical ultracentrifugation of PRL proteins. Absorbance data color-coded from beginning of the run (*purple*) to the end (*red*) are shown along with the fits and residuals from the SEDFIT analysis.

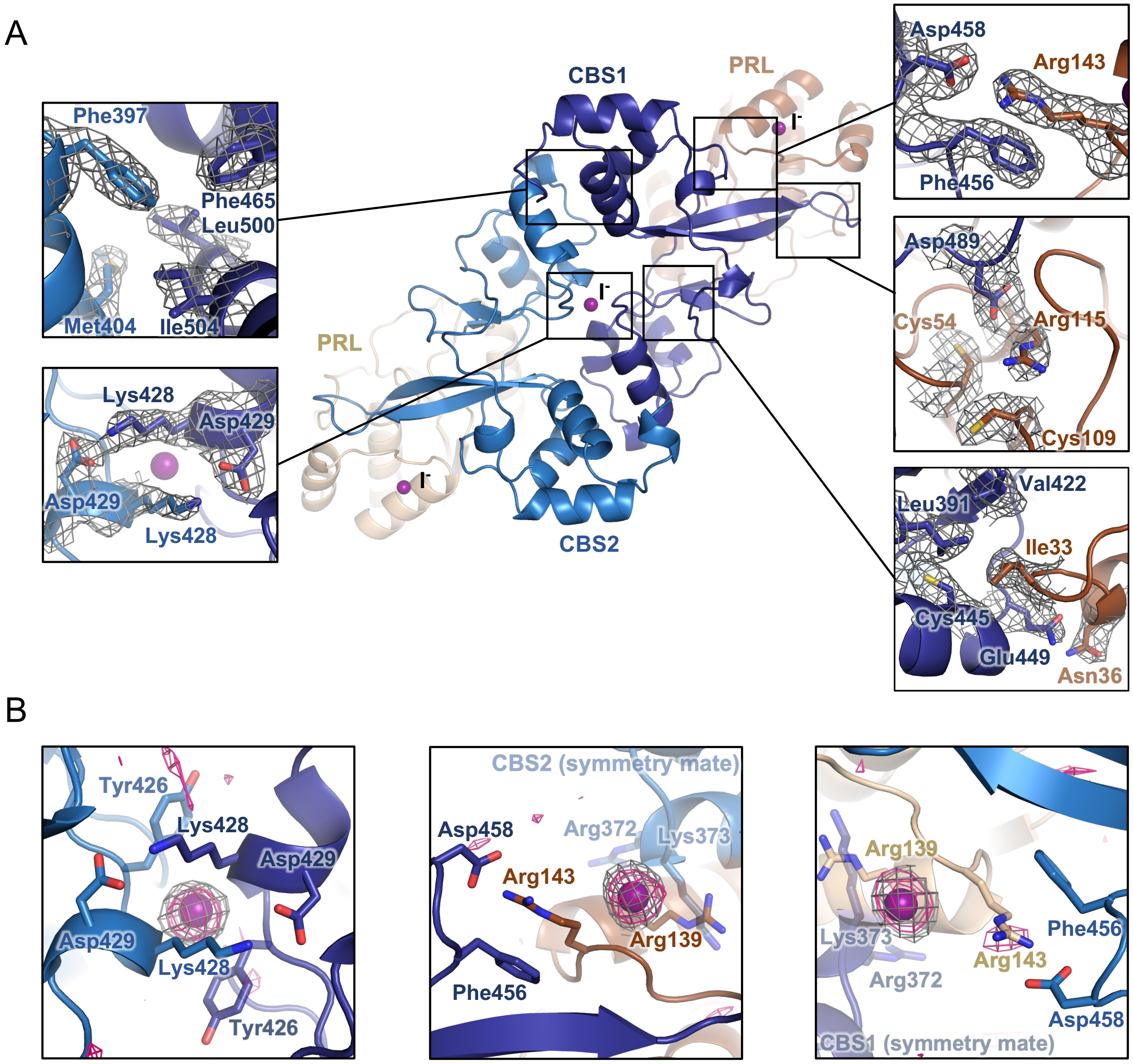

Figure S3. Electron density map of fly PRL/CBS crystal structure elements. (A) 2Fo-Fc omit map (grey) of residues involved in protein-protein contacts between the fly CBS-pair domains, and between the fly CBS-pair domain and fly PRL protein. **(B)** Anomalous difference map (magenta) of the three iodides in the crystal structure, overlayed with a 2Fc-Fc omit map. Iodide ions bind primarily at hydrophobic sites and positively charged sites (near arginines and lysines) in the crystal. Contour levels of 2Fo-Fc and anomalous difference maps are set to 1 RSMD and 3 RMSD, respectively.

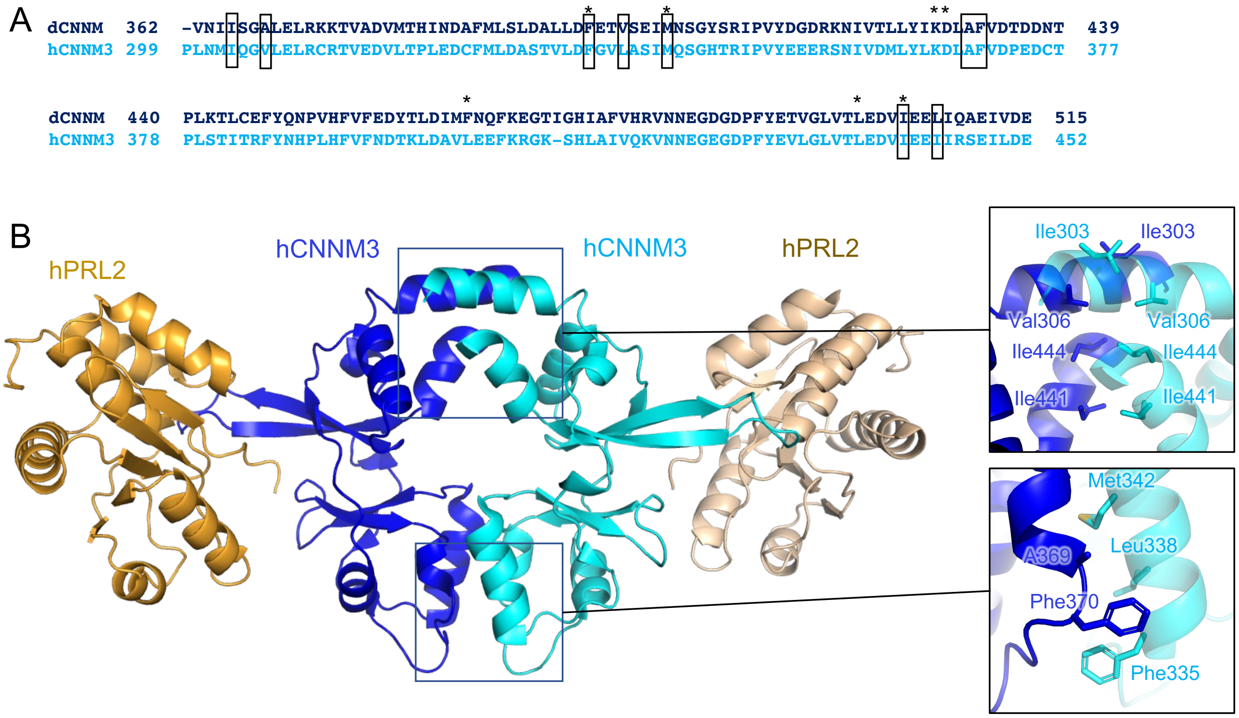

Figure S4. Dimerization of the CBS-pair domain in human CNNM3.(A) Sequence alignment of the CBS-pair domain of human CNNM3 and *Drosophila* CNNM. Residues involved in conventional head-to-head parallel dimerization are framed, whereas those involved in head-to-tail anti-parallel dimerization are indicated with an asterisk (*). **(B)** Crystal structure of the human complex of PRL2 and the CBS-pair of CNNM3 (PDB entry 5K22). Details of the protein-protein contacts involved in head-to-head parallel dimerization of the CBS-pair domain are shown on the right.

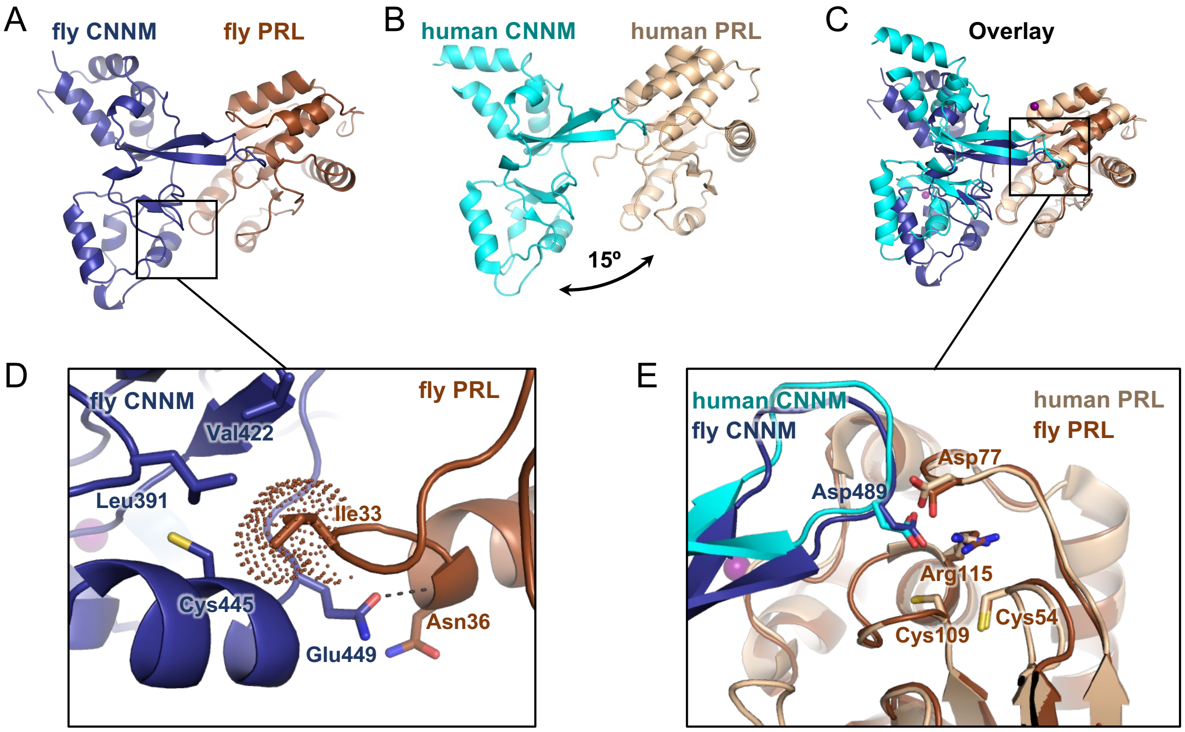

Figure S5. Comparison of fly and human PRL/CBS-pair structures. (A) Structure of *Drosophila* PRL-CNNM complex. **(B)** PRL2-CNNM3 structure (PDB: 5K22) showing the larger contact angle between the proteins in the human complex. **(C)** Overlay of the fly and human complexes. **(D)** Detail of the contacts between PRL helix α1 and the CBS-pair domain. PRL Ile33 fits into a hydrophobic pocket formed by CNNM Leu391, Val422, and Cys 445. Electrostatic stabilization is provided by the aligned α-helical dipoles and a hydrogen bond between Glu449 and the backbone amide of PRL Asn449. **(E)** Detail of the overlay of the fly and human complexes, showing the structural and sequence conservation. Residues are numbered according to the fly sequences.

Literature cited

1. Waterhouse, A. M., Procter, J. B., Martin, D. M. A., Clamp, M., and Barton, G. J. (2009) Jalview Version 2—a multiple sequence alignment editor and analysis workbench. *Bioinformatics (Oxford, England)* **25**, 1189-1191
2. Sievers, F., and Higgins, D. G. (2018) Clustal Omega for making accurate alignments of many protein sequences. *Protein Sci* **27**, 135-145
